# The Global and Local Distribution of RNA Structure throughout the SARS-CoV-2 Genome

**DOI:** 10.1128/JVI.02190-20

**Published:** 2021-02-10

**Authors:** Rafael de Cesaris Araujo Tavares, Gandhar Mahadeshwar, Han Wan, Nicholas C. Huston, Anna Marie Pyle

**Affiliations:** a Department of Chemistry, Yale University, New Haven, Connecticut, USA; b Department of Molecular Biophysics and Biochemistry, Yale University, New Haven, Connecticut, USA; c Department of Molecular, Cellular and Developmental Biology, Yale University, New Haven, Connecticut, USA; d Howard Hughes Medical Institute, Chevy Chase, Maryland, USA; University of Texas Southwestern Medical Center

**Keywords:** SARS-CoV-2, RNA structure, base pair content, structural stability

## Abstract

Severe acute respiratory syndrome coronavirus 2 (SARS-CoV-2) is the causative agent of COVID-19, the disease at the center of the current global pandemic. While knowledge of highly structured regions is integral for mechanistic insights into the viral infection cycle, very little is known about the location and folding stability of functional elements within the massive (∼30-kb) SARS-CoV-2 RNA genome. In this study, we analyzed the folding stability of this RNA genome relative to the structural landscape of other well-known viral RNAs. We present an *in silico* pipeline to predict regions of high-base-pair content across long genomes and to pinpoint hot spots of well-defined RNA structures, a method that allows for direct comparisons of RNA structural complexity within the several domains in SARS-CoV-2 genome. We report that the SARS-CoV-2 genomic propensity for stable RNA folding is exceptional among RNA viruses, superseding even that of hepatitis C virus (HCV), one of the most structured viral RNAs in nature. Furthermore, our analysis suggests various levels of RNA structure across genomic functional regions, with accessory and structural open reading frames (ORFs) containing the highest structural density in the viral genome. Finally, we took a step further to examine how individual RNA structures formed by these ORFs are affected by the differences in genomic and subgenomic contexts, which, given the technical difficulty of experimentally separating cellular mixtures of subgenomic RNA (sgRNA) from genomic RNA (gRNA), is a unique advantage of our *in silico* pipeline. The resulting findings provide a useful roadmap for planning focused empirical studies of SARS-CoV-2 RNA biology and a preliminary guide for exploring potential SARS-CoV-2 RNA drug targets.

**IMPORTANCE** The RNA genome of SARS-CoV-2 is among the largest and most complex viral genomes, yet its RNA structural features remain relatively unexplored. Since RNA elements guide function in most RNA viruses, and they represent potential drug targets, it is essential to chart the architectural features of SARS-CoV-2 and pinpoint regions that merit focused study. In this study, we found that RNA folding stability of SARS-CoV-2 genome is exceptional among viral genomes and we developed a method to directly compare levels of predicted secondary structure across SARS-CoV-2 domains. Remarkably, we found that coding regions display the highest structural propensity in the genome, forming motifs that differ between the genomic and subgenomic contexts. Our approach provides an attractive strategy to rapidly screen for candidate structured regions based on base pairing potential and provides a readily interpretable roadmap to guide functional studies of RNA viruses and other pharmacologically relevant RNA transcripts.

## Abstract (precis)

The RNA genome of SARS-CoV-2 is among the largest and most complex viral genomes, yet its RNA structural features remain relatively unexplored. Since RNA elements guide function in most RNA viruses, and they represent potential drug targets, it is essential to chart the architectural features of SARS-CoV-2 and pinpoint regions that merit focused study.

## INTRODUCTION

Severe acute respiratory syndrome coronavirus 2 (SARS-CoV-2) is an enveloped positive-strand RNA virus and the etiological agent of COVID-19 (1), a highly infectious human disease at the center of a worldwide pandemic (2–4). This virus is a member of the coronavirus family, known for having the largest genomes among all RNA viruses (5). Almost 30 kb in length (6), the SARS-CoV-2 RNA genome presents new challenges to RNA structural biology due to its size and complexity.

Following viral entry and uncoating, the genomic RNA serves as the template for translation of a multicomponent replicase-transcriptase complex that is responsible for synthesizing the viral transcriptome, which includes a series of subgenomic RNAs from which other virion components and accessory protein factors are expressed (5). Consistent with reports on other coronaviruses, the SARS-CoV-2 genome contains highly conserved RNA structural elements that likely play pivotal roles in viral replication, including several structures in the untranslated regions (UTRs) and a ribosomal frameshifting element (7). Although some of these motifs have been functionally studied and modeled in other betacoronaviruses (8–11), little is known about functional structural elements in the overwhelming majority of regions within the SARS-CoV-2 genome.

In line with previous reports on other coronaviral genomes (12), SARS-CoV-2 was recently suggested to form a genome-scale ordered RNA structure (GORS) (13, 14). As shown in foundational work comparing several families of RNA viruses (12) and further explored in later studies (15–17), the existence of GORS in positive-strand RNA viruses correlates with features like fitness and persistence. These studies have also established hepaciviral genomes as textbook examples of globally structured RNAs, and the most studied member of this genus, hepatitis C virus (HCV), is among the most highly structured viral RNAs characterized to date. The abundant RNA structures found throughout the (mostly) coding regions of that genome not only play individual functional roles (18–20) but also contribute to its higher-order compaction (15).

In light of the pervasive importance of RNA structural elements in the life cycle of RNA viruses, it is essential to understand the relative distribution of RNA secondary structure in the SARS-CoV-2 genome on both global and local scales. A particularly useful way to evaluate the “structuredness” of a viral RNA genome is to compare its global folding stability to that of well-studied RNA sequences using minimum free energy Z-scores (12). In this study, we used this approach to evaluate SARS-CoV-2 secondary structural stability relative to other structured viral genomes and also globally unstructured RNAs. Inspired by this approach, we adapted this strategy to identify and compare local regions of high base pair content (BPC) across long genomes. By applying this strategy to SARS-CoV-2, we obtained a comprehensive roadmap for the overall structural organization of the genome and the subgenomes, providing a guide for designing experimental strategies to explore the role of these elements *in vivo*.

Here, we show that the potential for stable RNA folding of the SARS-CoV-2 genome supersedes even that of HCV and discuss the potential biological consequences of this unprecedented level of global structural complexity. We developed a convenient pipeline to analyze the base pair content of any long RNA and to rapidly identify regions with predicted well-defined structure across kilobase transcripts. We used this pipeline to scan the SARS-CoV-2 genome and pinpoint regions with a high propensity to form stable secondary structures, enabling direct comparisons of structural content among the functional domains of this massive viral genome. We observed a remarkable enrichment of structured regions within open reading frames (ORFs) that encode accessory and structural proteins, and we elaborate on the potential roles these structures might play in the course of viral infection. Finally, we demonstrate that SARS-CoV-2 ORFs can adopt different structures in the genomic and subgenomic contexts.

The methods described in the present work enable investigators to extract base pair content information from any RNA structural model, including both *in silico* predictions and experimental chemical probing experiments. While we illustrate the utility of this approach by predicting stable architectural features within the SARS-CoV-2 genome, the pipeline can be implemented for characterizing the architectural landscape of any long RNA, and it is particularly valuable during early stages of discovery, when little is known about a virus or transcript. Therefore, our pipeline complements and guides parallel experimental approaches for identifying regulatory and therapeutic targets (21).

## RESULTS

### The SARS-CoV-2 genome contains an unprecedented level of stable RNA structure.

**FIG 1:**
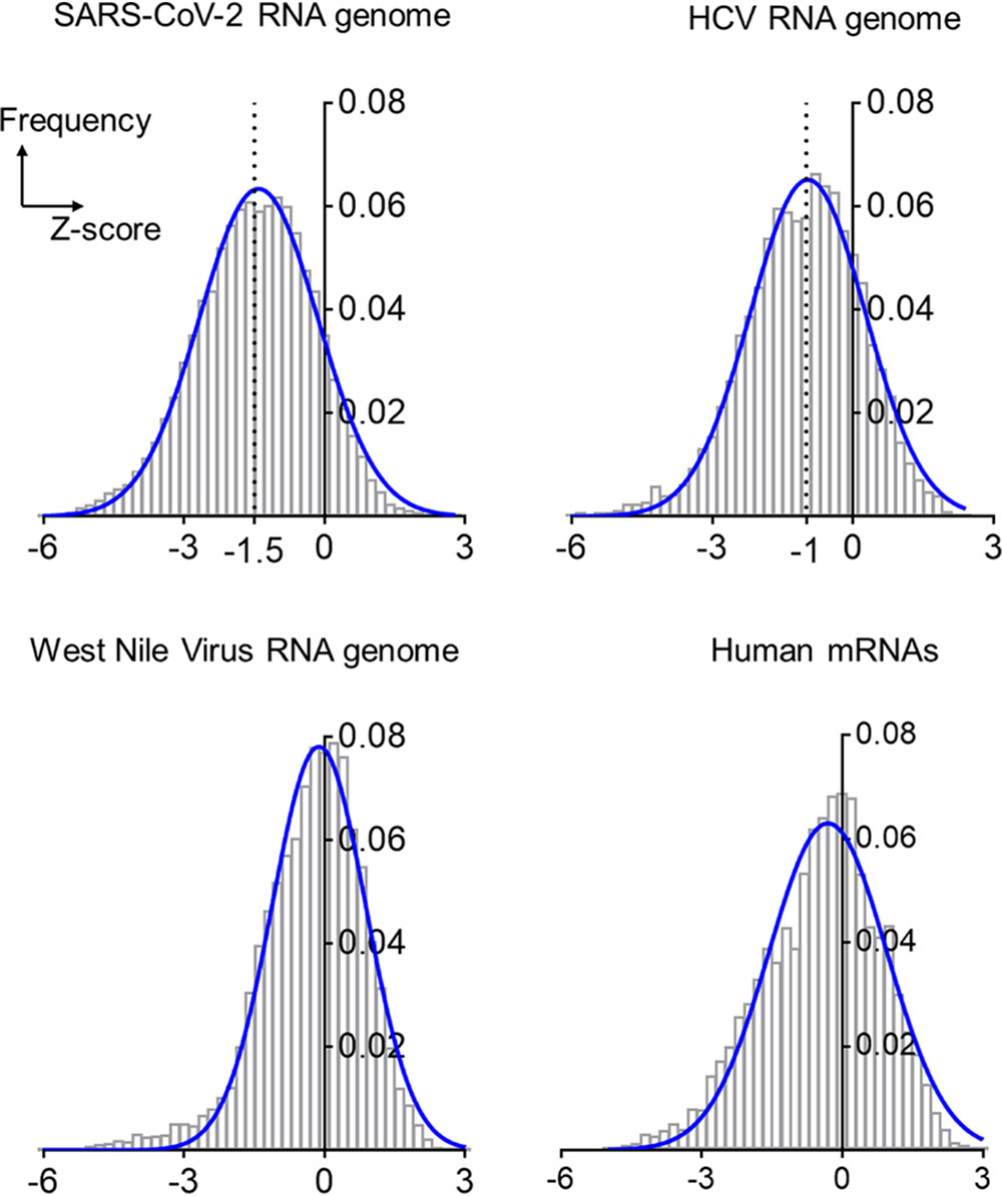
Distributions of Z-scores for the RNA genomes of SARS-CoV-2, HCV, and West Nile viruses and a composite of human mRNAs. The bar plots are frequency distributions (*y* axis) of free-energy Z-scores (*x* axis) calculated in sliding windows tiling each RNA. Each histogram is overlaid with a Gaussian (normal distribution) fit represented by a solid blue curve.

As an initial global approach to evaluate SARS-CoV-2 RNA structural stability, we used ScanFold (22) to calculate free-energy Z-scores in windows that were tiled along the entire genome (see Materials and Methods) and analyzed their frequency distribution. In parallel, we performed the same analysis with the HCV genome, which is a hallmark example of globally structured viral RNA and one of the most highly structured RNA genomes ever characterized (12, 19, 23). West Nile virus was also included for comparison, as viruses in the *Flavivirus* genus are thought to lack globally ordered RNA genomes (12). Finally, we analyzed a composite set of human mRNAs as a nonviral control believed to lack extensive internal RNA structure (Fig. 1).

As anticipated, the human mRNAs sampled showed little global tendency to form stable RNA structures (median Z-score, −0.35 [Fig. 1]), which is consistent with the presence of local UTR structures and relatively low levels of structure along open reading frames (24, 25). In the case of the West Nile virus genome, a Z-score distribution centered at −0.2 (median) similarly suggests the absence of globally ordered RNA folding, in agreement with trends observed for other *Flavivirus* RNA genomes (12) and also for RNAs originated from the genomes of double-stranded DNA (dsDNA) viruses like human herpesviruses (26). In very local regions, the human mRNAs and flaviviral genome cases displayed a low frequency of highly negative Z-scores (e.g., values below −3, which indicate the presence of highly stable RNA secondary structures), but both distributions suggest the absence of widespread base pairing. In contrast, Z-score distribution for the HCV genome was dominated by negative values (median Z-score, −1 [Fig. 1]), indicating a genome-wide propensity to form stable RNA base-pairings. This observation agrees with previous genome-wide analyses of HCV structural content (12) and studies of discrete RNA secondary structures throughout the HCV UTRs and coding regions (19, 20, 27, 28).

The Z-score distribution for the SARS-CoV-2 genome is shifted far into the negative range (Fig. 1), indicating that the genome has a much greater propensity to form stable secondary structures than other RNAs analyzed, by far more than is possible by chance. This is consistent with the reported preference for ordered folding seen in some coronaviral RNAs, like that of mouse hepatitis virus (MHV) (12). Most strikingly, the SARS-CoV-2 Z-score distribution is centered about a significantly more negative value (median Z-score, −1.5) than observed for HCV, suggesting that the SARS-CoV-2 genome has almost twice the propensity to form stable base pairings than one of the most structured RNA genomes in nature and that it is likely to form extensive secondary structures throughout all of its functional domains, in both coding and noncoding regions. This unusual level of RNA structural stability suggests a vast network of functional RNA structures within the SARS-CoV-2 genome.

### A versatile pipeline for quantifying base pair content within an RNA genome.

**FIG 2:**
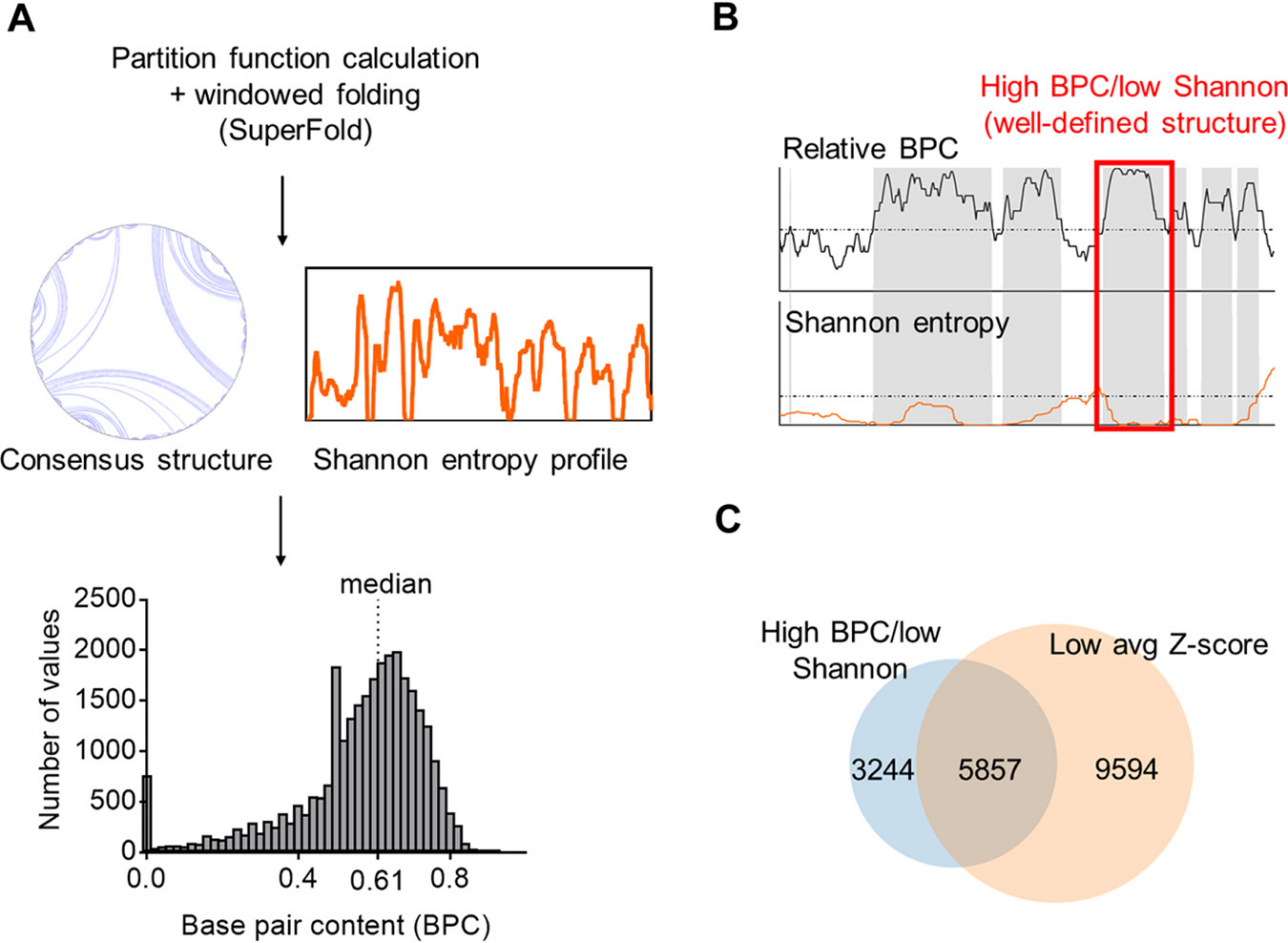
A pipeline to predict and quantify the base pair content across SARS-CoV-2 genome and identify well-defined structured regions. (A) A scheme depicting the steps to predict the secondary structure of SARS-CoV-2 genome in windows using SuperFold. A histogram of base pair content (BPC) values calculated from the predicted secondary structure (gray bar plot) is shown, and the median BPC is indicated (0.61). (B) A strategy to identify well-defined structures. The scheme shows shaded regions containing nucleotides that pass two criteria: high relative BPC (upper graph, dashed line indicating the median value of 0.5) and low Shannon entropy (lower graph, dashed line indicating the global Shannon median). The red square highlights one of the regions flagged as forming a well-defined structure. (C) A Venn diagram showing the overlap between the total number of nucleotides identified as having well-defined structure using the procedure for panel B and those nucleotides with low average Z-scores (below the global median) as reported by Andrews et al. (13).

To map and visualize the entire SARS-CoV-2 RNA structural network, we developed a pipeline for quantitating and comparing relative levels of base pair content (BPC) and secondary structural features throughout the genome (Fig. 2). Initially, we used SuperFold (29) to fold the 29.9-kb genome of SARS-CoV-2 in overlapping windows, enabling us to compute a preliminary full-length secondary structure and a genome-wide Shannon entropy profile derived from base pairing probabilities. We then used the resulting secondary structure to calculate the BPC by scanning the entire RNA in sliding windows (see Materials and Methods). We found that the SARS-CoV-2 genome is predominantly folded into discrete secondary structural motifs that are predicted to have high thermodynamic stability, with an average BPC of 61% (Fig. 2A, median value indicated). This finding agrees with the Z-score analysis, which indicated a global propensity for stable structural folding (Fig. 1). In order to directly compare the relative structural contents of different regions, we also quantified the relative base pair content (BPC_rel_) for each section across the SARS-CoV-2 genome (Fig. 2B). We define BPC_rel_ as the percentile of BPC at a given site relative to the overall BPC distribution along the length of the RNA (see Materials and Methods).

We then proceeded to sift through the genome, locating discrete regions of well-determined RNA secondary structures. To accomplish this, we adapted a motif discovery method that was originally developed for interpreting the results of chemical probing experiments (29). But instead of using SHAPE reactivities as an input, we used the BPC_rel_ distribution across the SARS-CoV-2 genome in conjunction with the corresponding Shannon entropy profile (scheme shown in Fig. 2B). This enabled us to flag regions with BPC_rel_ values above 0.5 (i.e., representing BPC values above the predicted global median) and correlate them with Shannon entropy values below the global median, resulting in a metric we define as “high BPC/low Shannon” (see Materials and Methods). This definition, which is analogous to the “low SHAPE/low Shannon” designation for flagging probable regions of uniquely determined secondary structure (29), reveals that 9,101 nucleotides, or a third of the entire genome, are located in regions of both high BPC and low Shannon entropy. Since nucleotides with low Shannon entropy are likely to favor a single, well-defined folding state (30), high-BPC/low-Shannon regions are, therefore, clusters of well-defined structures with potential functionality. Our analysis therefore suggests a remarkable abundance of well-defined secondary structures within the SARS-CoV-2 genome.

In order to assess the relative thermodynamic stability of specific structured regions defined by this approach, we calculated the relative enrichment in stable base pairs as defined by the ScanFold-Fold analysis in the work of Andrews et al. (13) and computed the overlap between the two approaches (Fig. 2C). Importantly, we observed that 64% of high-BPC/low-Shannon-entropy regions overlap with regions that have low average Z-scores (also defined here relative to the overall median) and that this enrichment is statistically significant (*P* value < 1e−05; see Materials and Methods). In this way, we confirmed that the SARS-CoV-2 RNA structural network has a high level of thermodynamic stability.

### The SARS-CoV-2 genome contains specific loci of well-defined RNA structures.

**FIG 3:**
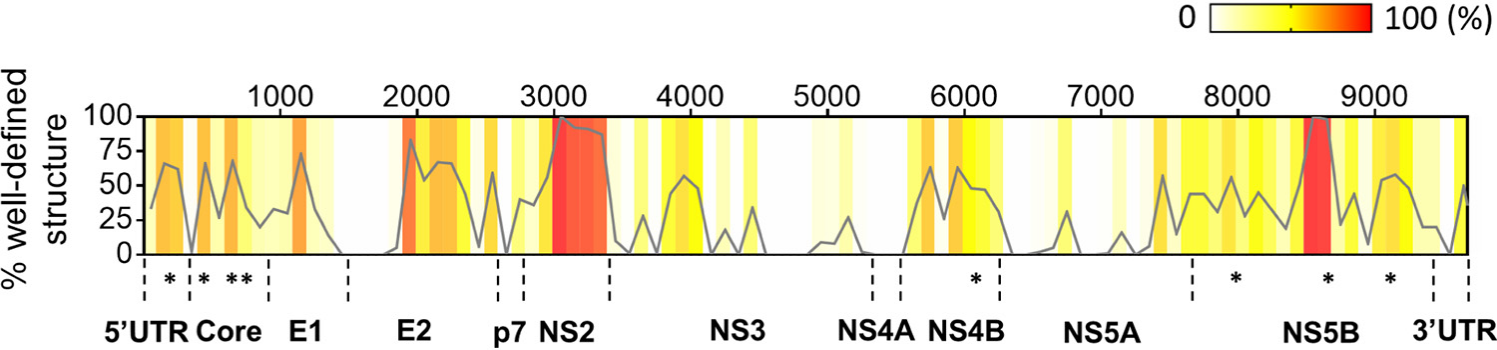
Distribution of well-defined RNA structures predicted for the HCV genome. The percentage of nucleotides in well-defined structured regions (high BPC/low Shannon) was calculated in 100-nt bins tiling HCV genomic sequence and is plotted as a function of the genomic coordinate (gray curve). Individual percentages of each genomic bin are also represented as a heatmap in the graph (color legend on the top right-hand corner). The locations of well-studied structural elements in the HCV genome are indicated with asterisks next to their respective genomic divisions, and details on each individual element are presented in Table 1.

Given the abundance of secondary structural units that correlate with low Shannon entropy values in SARS-CoV-2 (high BPC/low Shannon, as defined in Fig. 2), we were interested in mapping their distribution across the genome and correlating their location with other units of genomic architecture. Before embarking on this strategy, we evaluated the methodology on a viral transcript that is reasonably well characterized. To this end, we computed the predicted distribution of well-defined structures (i.e., high BPC/low Shannon) in the (+) genomic RNA of hepatitis C virus (HCV; JC1 strain). We found that several genomic domains in HCV are enriched with well-defined structures as defined by our approach, including the 5′ UTR and ORF regions encoding the Core structural protein and nonstructural proteins NS4B and NS5B (Fig. 3), which are known to harbor stable, functionally validated RNA structural elements (19, 23, 31). We observed that several of these elements overlap with regions of high structural content across their genomic segments, and we predict novel structures in regions of HCV that have not been the focus of prior investigation (Fig. 3 and Table 1). These results suggest that our strategy can be used to accurately scan kilobases of RNA sequence for candidate RNA structural elements that merit downstream investigation, which is particularly valuable for viral transcripts of unknown structural composition.

**TABLE 1:** Well-studied RNA structural elements in the HCV genome

RNA structural element (reference[s])	Sequence interval (HCV JC1)	Genomic domain
IRES (65)	1–350	5′ UTR
SL388 (19, 66), SL427 (19, 67)	417–488	Core
SL588, SL669 (19, 67)	588–749	Core
J750 (19, 23, 67)	751–824	Core
SL6038 (19)	6038–6186	NS4B
J7880 (23)	7880–7998	NS5B
SL8670 (23)	8655–9716	NS5B
SL9074 (68, 69), SL9198 (70)	9103–9257	NS5B

**FIG 4:**
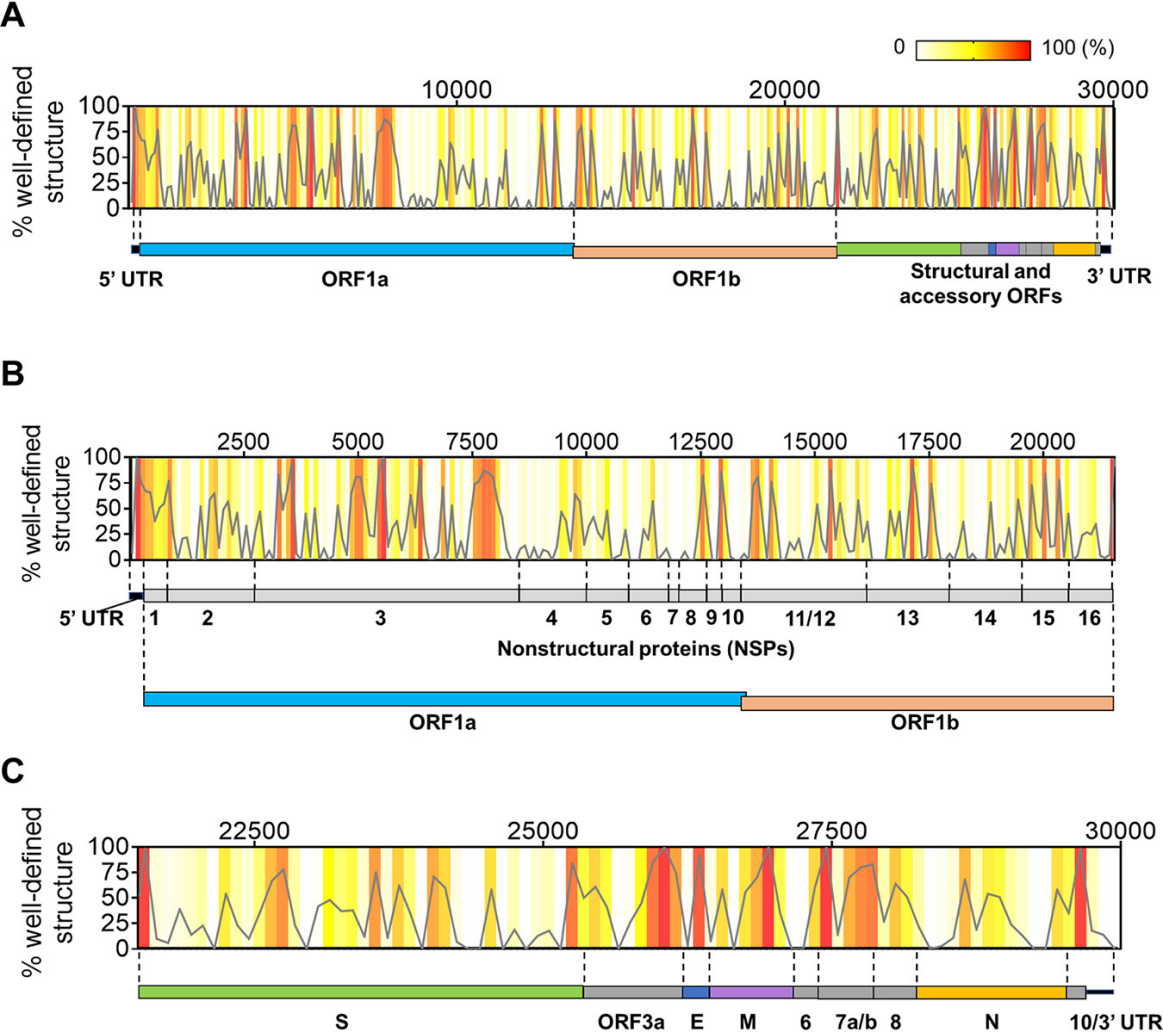
Distribution of well-defined RNA structures across the SARS-CoV-2 genome. (A) The percentage of nucleotides in well-defined structured regions (high BPC/low Shannon) was calculated in 100-nt bins tiling the genome and is plotted as a function of the genomic coordinate (gray curve). Individual percentages of each genomic bin are also represented as a heat map in the same graph (color key on the top right-hand corner). A scheme representing the genomic divisions of SARS-CoV-2 is shown next to the plot to guide location of structured regions. (B) An expanded view of the initial two-thirds of the genome from the graph in panel A is shown along with the genomic divisions of this region (UTR plus ORF1ab and corresponding NSP divisions). (C) The downstream third of the genome is expanded from the graph in panel A to zoom in on individual structural and accessory ORFs in this region.

**FIG 5:**
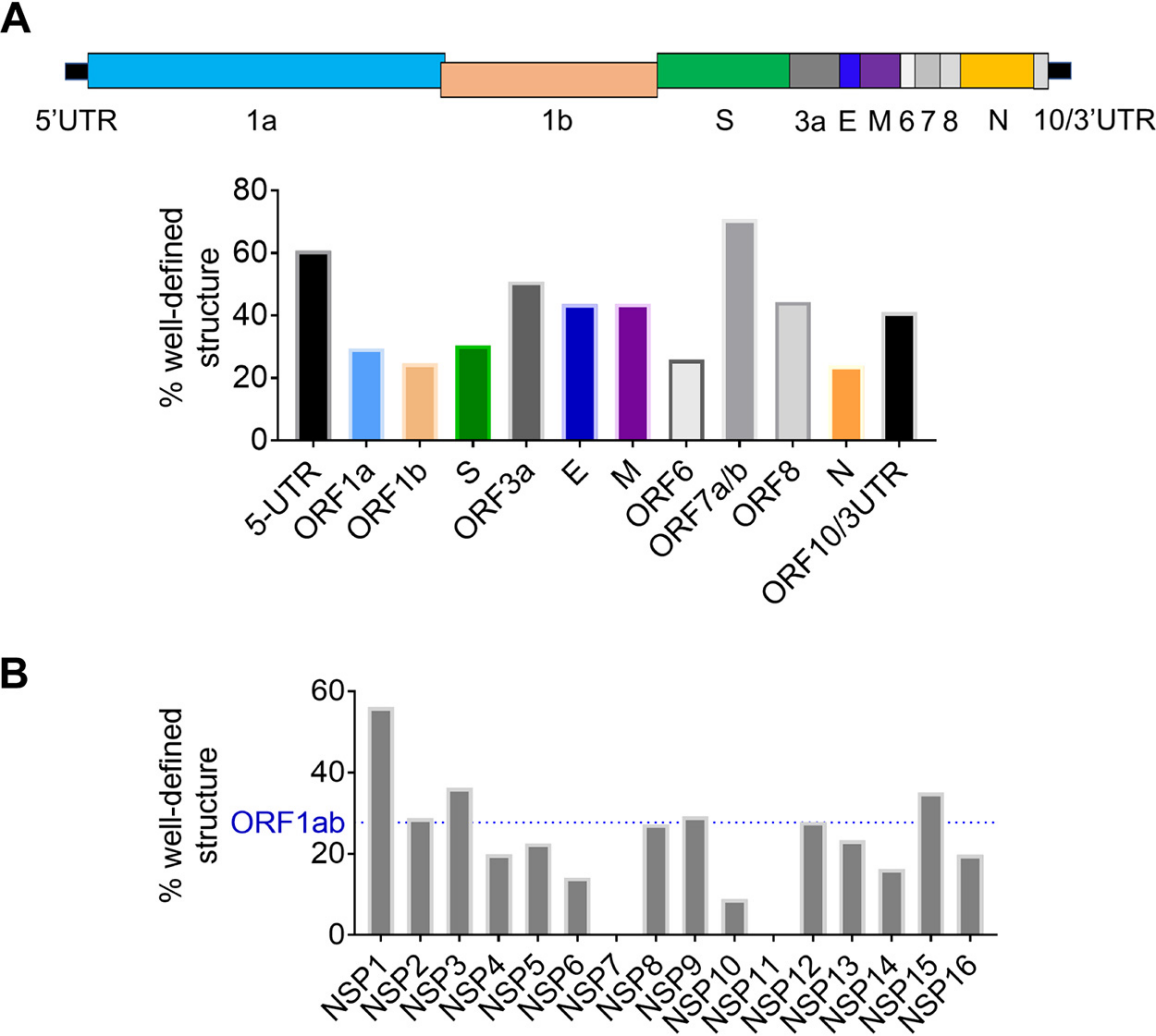
Quantification of well-defined structure in SARS-CoV-2 subdivisions. (A) The percentages of nucleotides with well-defined structure (high BPC/low Shannon) are shown for each genomic section of SARS-CoV-2. A cartoon of genomic regions is depicted above the bar plot, and each region is color coded relative to the bar graph. (B) The percentages of nucleotides with well-defined structure (high BPC/low Shannon) are shown (gray bars) for each NSP (nonstructural protein) section of SARS-CoV-2 ORF1ab. The horizontal dashed line (blue) represents the percentage corresponding to the entire ORF1ab.

We then calculated the fraction of high-BPC/low-Shannon nucleotides in bins that tile the SARS-CoV-2 genome, resulting in a genome-wide distribution of well-defined structures that can be plotted as a function of position along the RNA. This distribution can be represented as a heat map of well-defined structures along the full-length SARS-CoV-2 genome (Fig. 4A), with expanded views of the initial two-thirds (5′ UTR and ORF1ab [Fig. 4B]) and the downstream one-third of the RNA (structural/accessory ORFs and 3′ UTR [Fig. 4C]). Various degrees of structural content are predicted across the genome, ranging from 24% to 71% of well-defined structures within individual domains (Fig. 5A).

To assess how these structured regions are distributed throughout the genomic RNA, we started by analyzing the noncoding regions. As expected, we found a high level of structural content in each of the untranslated regions (the 5′ and 3′ UTRs): indeed, 61% of the 5′ UTR and 41% of the 3′ UTR are characterized by well-defined structures (Fig. 4A and Fig. 5A), which is consistent with the presence of RNA regulatory elements that play roles in replication and translation of the virus.

However, unlike conventional mRNAs or flaviviral RNAs, RNA structural elements are not predominantly confined to the UTRs, as they are observed throughout all coding regions of SARS-CoV-2 (Fig. 4A). Different regions of the ORF contain various amounts of secondary structure. For example, we observe that 27% of ORF1ab contains nucleotides within high-BPC/low-Shannon regions, which are spread sparsely over more than 21 kb (∼2/3 of the genome). These foci of well-defined RNA structures are not uniformly distributed along this ORF, as individual NSP domains contain vastly different degrees of secondary structure (Fig. 4B and Fig. 5B). The most upstream segment, NSP1, is the most structured region of ORF1ab, with 56% of its nucleotides forming well-defined motifs (Fig. 5B). Importantly, the upstream half of the NSP1 segment appears to be part of a large module that forms in conjunction with the 5′ UTR, as a peak of high BPC/low Shannon values encompasses both domains (Fig. 4B). This suggests that, as observed in other coronaviruses (11), upstream regulatory elements of the genome extend far into the ORF. The largest domain in ORF1ab, NSP3, contains highly structured foci that can be organized into three big clusters (Fig. 4B): one cluster is located adjacent to the 5′ terminus of the domain (nucleotides [nt] 3200 to 3600), a middle section displays multiple high-BPC/low-Shannon peaks (nt 4500 to 6500), and a downstream segment is located near the 3′ NSP3 terminus (nt 7450 to 8200). This overall organization suggests that NSP3 contains independent modules of RNA secondary structure. Similar clusters of RNA structures are observed in NSP12 and -13, and they occur roughly within the limits of each domain. In contrast, other NSP regions (NSP4, -5, -6, -8, -9, -10, -14, -15, and -16) form structures that encompass the boundaries of individual segments, suggesting a modular organization at the RNA level that does not necessarily correlate with functionality at the protein level. Finally, we observed that regions corresponding to NSP7 and NSP11 showed a complete absence of well-determined structures (Fig. 5B), thereby suggesting the presence of predominantly unstructured regions in the genome.

The downstream third of the SARS-CoV-2 genome, which contains ORFs for structural and accessory proteins (the subgenomic RNA [sgRNA]-encoding region), displays a much higher overall secondary structural content than ORF1ab and has 36% of its sequence folding into well-determined structures (Fig. 4C). Remarkably, some of these ORFs (ORF3a, -E, -M, -7ab, and -8) have a predicted structural content that is comparable to or even higher than that of the UTRs (Fig. 5A), with the most prominent example being ORF7ab (high BPC/low Shannon fraction of 70%). These highly structured ORFs are all relatively short (ranging from 236 to 852 nt), consisting of a series of well-defined structures that are very closely spaced (Fig. 4C). On the other hand, longer ORFs like S (spike) and N (nucleocapsid) contain shorter patches of well-defined structures interspersed with longer, less structured regions, resulting in a somewhat lower structural content for these ORFs (30% for the S ORF and 24% for the N ORF [Fig. 5A]).

Similar to patterns observed for ORF1ab, we also observed RNA structural modules that span multiple ORFs. One example is a module that spans the junction between S ORF and ORF3a, including the transcription regulatory sequence (TRS) at the intersection between them. Similarly, part of ORF6 folds into a substructure that includes elements of ORF7a/b and -8, resulting in an extended structured region that includes three TRS elements. These observations indicate that some TRSs in this region might engage in structures with their surrounding ORFs, a feature that is likely to influence sgRNA synthesis and replication. Taken together, these results suggest the formation of numerous modules of well-determined RNA structure throughout the SARS-CoV-2 genome and reveal important structural trends across its genomic sections.

**FIG 6:**
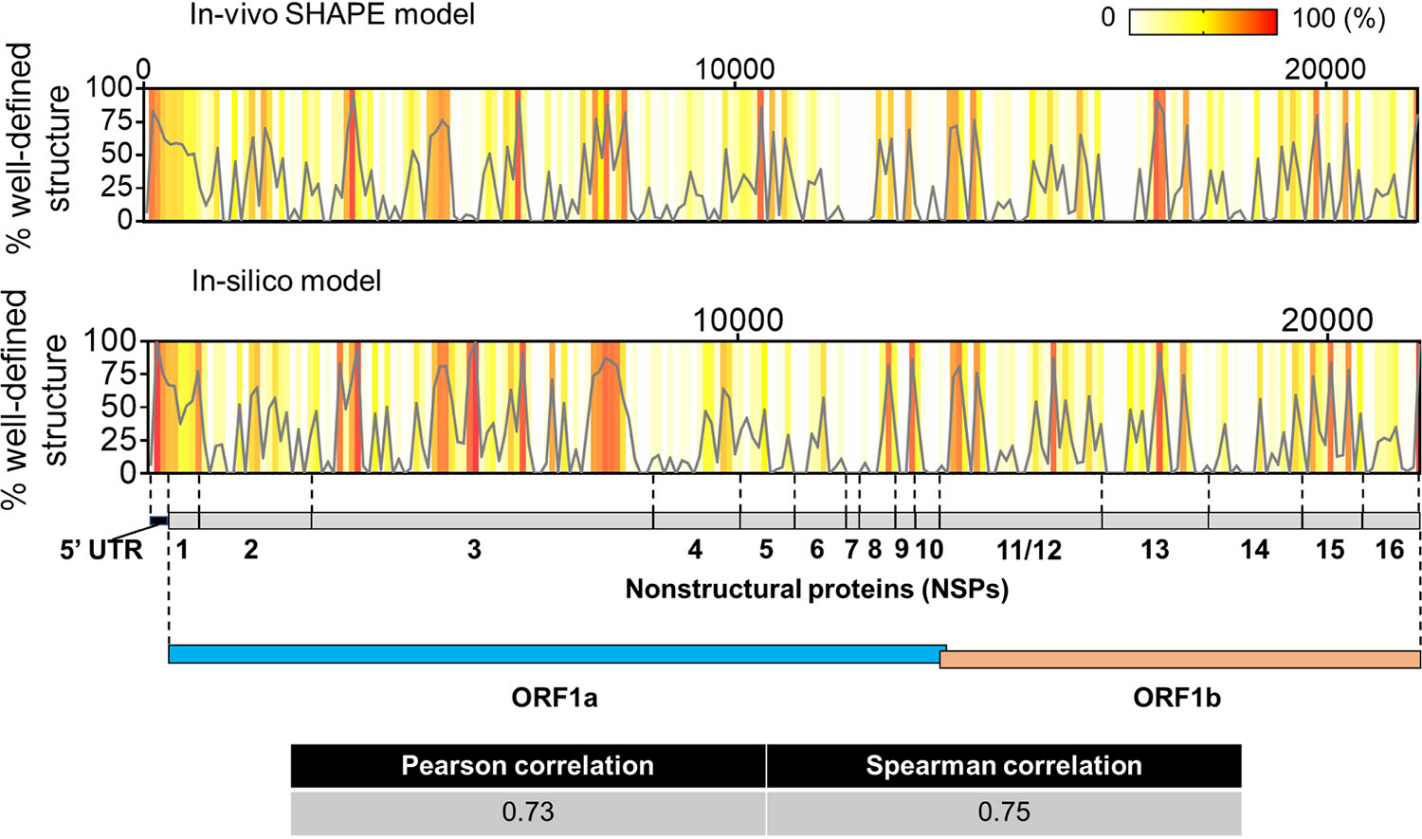
Comparison between *in silico* prediction in this study and experimental (in-cell SHAPE) structure reported by Huston et al. 2020 (32) for the ORF1ab region (including the 5′ UTR). The plots (gray lines) show the distribution of well-defined structure (percent high base pair content/low Shannon entropy) calculated in 100-nt bins tiling the ORF1ab region from both structural models. The same values are represented as a heat map in each graph to depict regions of high and low structural content according to the key shown on the upper right-hand corner. A cartoon with the genomic subdivisions of ORF1ab is shown to guide data visualization. The computed correlation coefficients between both data sets are shown in the table.

In order to evaluate the validity of our conclusions using an orthogonal data set, we applied the same computational pipeline to compute the fraction of high-BPC/low-Shannon-entropy nucleotides across ORF1ab (including the 5′ UTR) using experimentally determined chemical probing data obtained in infected cells (32). We then compared the resulting output with our purely *in silico* results (Fig. 6). Despite the marked differences expected between *in silico* and in cellulo settings, there is good overall agreement between both data sets (Pearson’s *R* = 0.73; Spearman’s rho = 0.75), which confirms the predicted distributions of well-defined structural hubs throughout the genomic subdivisions of ORF1ab. These results provide an additional experimental validation of the methods we describe here, extending them to applications using experimental data and suggesting broad applicability for characterizing structural trends in long viral RNAs.

### Secondary structural features depend on genomic versus subgenomic context.

Intrigued by the abundance of predicted RNA structures within the ORFs of SARS-CoV-2, we asked whether individual ORFs might adopt different structures depending on the context in which they are inserted, i.e., in genomic versus subgenomic RNAs. As a direct application of base pair content analysis, we calculated the predicted folded structure of one specific structural ORF that is present in both genomic RNA (gRNA) and subgenomic RNA. We chose the Nucleocapsid ORF because it forms the most abundant sgRNA (N sgRNA), which is estimated to be at least 1 order of magnitude more abundant than other sgRNAs (6).

**FIG 7:**
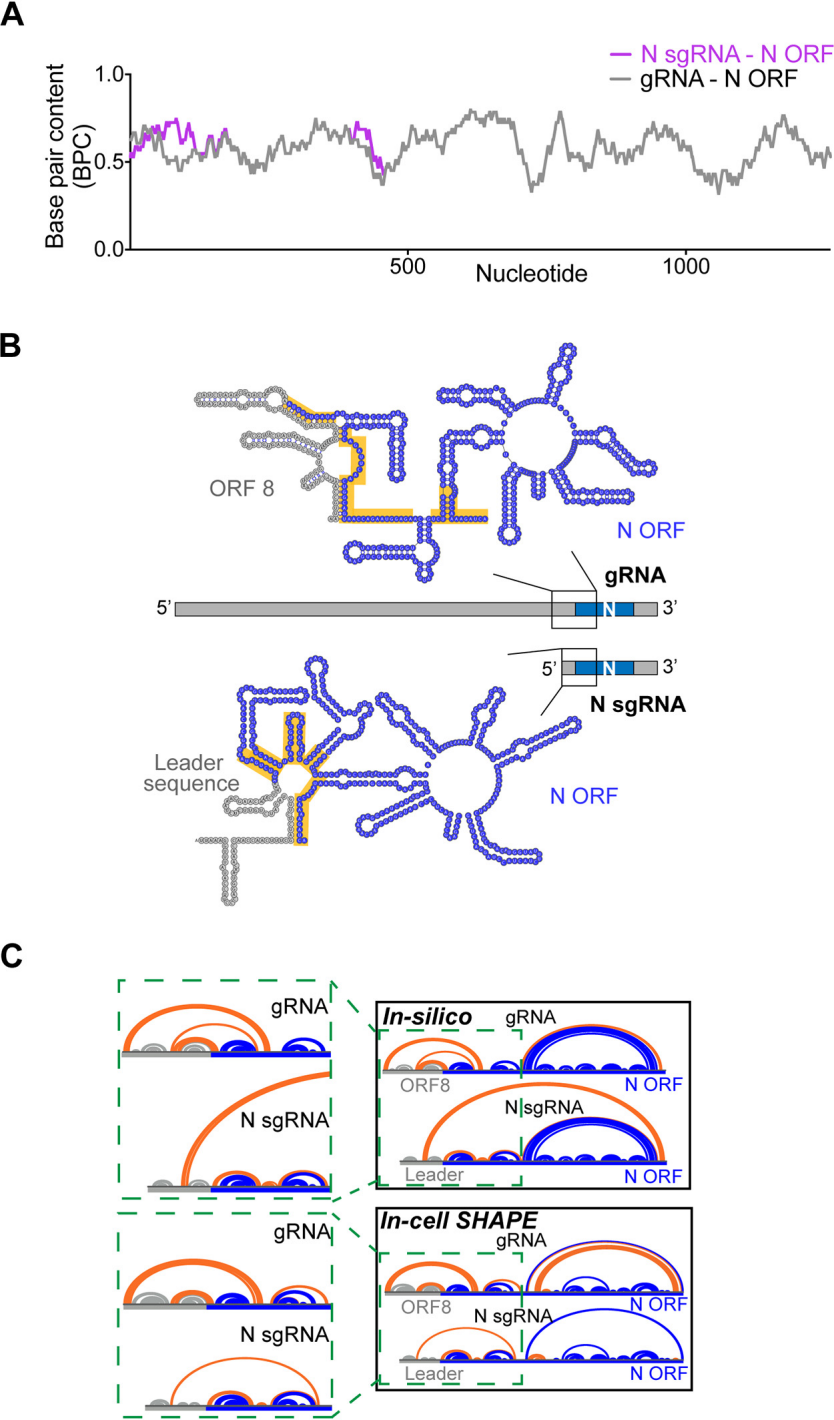
Context-dependent formation of secondary structures in the SARS-CoV-2 nucleocapsid ORF. (A) The base pair content for the N ORF (total of 1,260 nucleotides) is plotted as a function of the nucleotide number for the genomic RNA (gray curve) and the N sgRNA (magenta curve). The *x* axis numbering represents the N ORF nucleotide order (1 to 1260). (B) *In silico* secondary structure predictions containing the upstream 434 nucleotides of the N ORF are shown for both genomic and N subgenomic RNAs. The region containing structural differences identified in panel A is shown, and the highlighted regions (yellow) show significantly different RNA folding in both contexts. In the genomic RNA, the gray region represents a downstream segment of ORF8 and a 14-nt stretch of additional sequence containing the TRS (5′-ACGAAC-3′). In the N subgenomic RNA, the gray region is the 5′ leader sequence and a homologous stretch of additional sequence containing the TRS. Structures were drawn on VARNA (64). (C) Arc diagram comparison of *in silico* and in-cell SHAPE secondary structural models of upstream N ORF. Base pairs involving the N ORF that are context dependent (forming exclusively in either gRNA or sgRNA) are highlighted in orange. Base pairs forming within sequences upstream of the N ORF (ORF8 in gRNA or the leader sequence in N sgRNA) are represented in gray, and base pairs within the N ORF that are not affected by sequence context are drawn in blue. Green dashed boxes show expanded junction regions in both cases (ORF8-N ORF in gRNA, 5′ leader-N ORF in sgRNA).

When comparing the N ORF base pair content in both the genomic and subgenomic contexts (Fig. 7A), one observes subtle differences in the upstream segment of this ORF. Specifically, the upstream 434 nucleotides of N ORF show patches of significantly higher base pair content in the subgenomic context than in the genomic context. To understand this, we examined the specific predicted RNA secondary structure in both contexts (Fig. 7B). In the genomic context, sequences upstream of the N region (which belong to ORF8) are predicted to form base pairing interactions with the adjacent N ORF, resulting in a specific RNA structure that is uniquely dependent on the genomic environment. In contrast, in the subgenomic context, upstream regions of the N ORF are adjacent to the 5′ leader sequence, which folds somewhat autonomously into an independent motif and makes fewer contacts with the adjacent N sequences. As a result, upstream nucleotides of the N ORF form a compact alternative secondary structure in the sgRNA that is distinct and more thermodynamically stable and which has a higher overall BPC than the same sequence in the genomic context.

To test these theoretical predictions using experimental data, we isolated RNA from SARS-CoV-2 infected cells that had been treated with selective 2′-hydroxyl acylation analyzed by primer extension (SHAPE) reagents and then selectively amplified upstream regions of the N ORF in the sgRNA context and then separately in the gRNA context. In this way, it was possible to obtain experimentally determined secondary structural models for this same region of the N ORF in both distinct contexts and compare them with our *in silico* predictions (Fig. 7C). Strikingly, this analysis not only confirmed that upstream regions of the sgRNA ORF are indeed affected by their adjacent sequence context as predicted *in silico* but also confirmed the overall pattern of predicted secondary structure organization: in both theoretical and experimental models, the adjacent ORF8 sequence interacts much more extensively with N ORF nucleotides in the genomic context (orange gRNA arcs in Fig. 7C). In contrast, the 5′ leader sequence folds almost autonomously in the sgRNA, with the exception of a few poorly determined long-range contacts (high Shannon entropy), resulting in more extensive pairing among N ORF nucleotides in the sgRNA junction (orange sgRNA arcs in Fig. 7C). As a consequence, a more stable, compact structure forms at the upstream section of the N sgRNA than within its cognate region of the gRNA.

These results suggest that the same RNA sequences can adopt completely different structures in the subgenomic and genomic contexts, thereby potentially diversifying functionality of the viral genome, with significant implications for RNA stability, processing, and molecular mechanism. A compact structure with high BPC in the upstream segment of the N sgRNA might contribute to its stability as an independent transcript, potentially explaining the unusually high abundance of this sgRNA. It will be important to conduct genetic studies to assess this model and to perform analogous studies on the other sgRNA/gRNA combinations to evaluate how they might influence viral function. These limited studies exemplify the presence of context-dependent differences in the structures of specific viral RNA sequences, and they provide a framework for sifting through vast coronavirus genomes (and other genomes) to identify discrete elements of dynamic RNA secondary structure.

## DISCUSSION

The majority of biophysical and structural studies being conducted on coronaviruses focus on the viral proteins, such as the virion constituents and components of the replication-transcription machinery (33–36). However, RNA motifs within positive-strand RNA viruses guide many processes that are critical for the virus life cycle (19, 20, 27, 28, 37). SARS-CoV-2 is unlikely to be an exception to this rule, particularly given that, to our knowledge, it has the most elaborately structured RNA genome that has ever been reported to date, and many of its constituent structures are conserved across coronavirus families (7, 8, 10, 11). Until the present study was conducted, the genome of HCV was the landmark example of a highly structured viral RNA, and one of the most structured ORFs in nature, distinguished by networks of functionally essential RNA structural motifs throughout both the coding and noncoding regions (19, 23, 31). However, we report here that the SARS-CoV-2 genomic RNA is nearly twice as compact and structured as HCV based on its folding stability, even when adjusting for its vastly greater overall length (∼30 kb versus ∼10 kb). RNA structural motifs within the UTRs and ORFs of coronaviruses are seemingly larger and more complex than those observed in other virus families (38–41), suggesting that an understanding of coronavirus RNA structure will play a key role in understanding the mechanistic processes and vulnerabilities of this virus.

It is interesting to consider why the largest RNA genome might also be the most highly structured. One hypothesis is that the idiosyncratic SARS-CoV-2 genomic architecture serves a protective function. Biophysical studies have shown that extensively structured viral RNAs like HCV adopt highly condensed states in solution and that these are inaccessible to external probe hybridization (15), which has implications for primer design in viral test kits and for biological function. It is therefore reasonable to expect that the SARS-CoV-2 genomic RNA might adopt structural states that affect the way it interacts with viral, cellular, and exogenous factors. The architectural features of the massive SARS-CoV-2 genome may confer protection against cellular nucleases, which would facilitate sustained infection in cells. In addition, the folded architecture of the SARS-CoV-2 genome may enable it to hide in plain sight, reducing activation of host pattern recognition receptors (42). In other well-structured positive-stranded RNA viruses like HCV and murine norovirus (MNV), the formation of a genome-scale ordered RNA structure (GORS) correlates with decreased activation of antiviral pathways (16, 43). Cell-based assays have shown that highly structured viral transcripts have a reduced propensity to activate interferon responses compared with that of less structured viral RNAs (17). Understanding the interplay between the SARS-CoV-2 genome architecture and elements of the host immune system will undoubtedly be a rich area of future investigation.

Another consequence of the highly structured, compact SARS-CoV-2 RNA genome is that its reduced dimensionality would facilitate interactions between RNA structural elements that are otherwise far apart from one another in primary sequence. Bringing genomic elements into close spatial proximity of one another will support the formation of long-range interactions between distant segments of the genome. Much like the topologically associating domains (TADs) in the chromatin of eukaryotes (44), “RNA TADs” in viral genomes might have the capacity to control replication, translation, packaging, and many other processes, as suggested for structures that constrain ends of the HCV genome (45, 46). There is already precedent for this within the coronavirus family, as long-range contacts in the transmissible gastroenteritis virus (TGEV) genome and cross talk between the 5′ and 3′ ends of the MHV genome have been proposed to modulate aspects of sgRNA synthesis in each system (47, 48).

Given the many mechanistic implications for “structuredness” of the SARS-CoV-2 RNA genome, we were motivated to adapt and develop tools for quantifying overall base pair content, and motif stability, relative to the expanse of an entire genome. For example, to monitor the domain-level distribution of extensively base paired regions across this RNA, we developed a general strategy for extracting the base pair content from a secondary structure model using an approach that is readily applicable to any theoretical or experimentally determined RNA structure prediction. By further applying a Shannon entropy filter (30, 49), we were then able to focus our analysis on the regions of greatest base pairing propensity and well-determined secondary structural composition (high BPC/low Shannon). Downstream quantification of the density of high-BPC/low-Shannon nucleotides enabled us to generate a preliminary profile of their distribution along the entire RNA (Fig. 4), and we were able to use the same computational approach on experimental data to validate our *in silico* predictions and confirm the structural trends reported for ORF1ab (Fig. 6). In this way, we could rapidly map regions with high and low predicted RNA structural frequency along the SARS-CoV-2 genome, producing a snapshot of the structural landscape for this RNA and pinpointing areas that merit focused biophysical study. In massive RNAs, such as coronaviral genomes or certain eukaryotic mRNA transcripts, an approach that rapidly sifts information on structural content and puts it into a global and spatial context is vital for the discovery of regulatory modules and drug targets. The results and methods presented here will thus guide experimental approaches focusing on specific structures of SARS-CoV-2 genome, not only facilitating construct and primer design but also providing a tool to evaluate potential structural differences within the complex pool of RNAs produced during viral infection.

It is useful to reflect on the frequency and spatial distribution of secondary structures within the SARS-CoV-2 genome, as their placement along the genome is far from uniform. Our analysis predicts that ORFs in the downstream third of the genome contain the highest density of well-defined structures in the viral transcript. These ORFs encode the sgRNAs and the accessory and structural proteins that are required during later stages of replication (5, 6). One possibility is that more extensive RNA folding of downstream segments might increase their relative stability and safeguard them for later phases of viral infection. Importantly, several RNA structures in this segment encompass the transcription regulatory sequences (TRSs), which are key to production of the 3′-nested sgRNAs (50). Given that TRS elements mediate the fusion of each ORF terminus to the leader sequence during subgenomic replication, their structural context is likely to affect the frequency of template switching (leader-to-body fusion) at each fusion site, possibly involving interactions with the replicase-transcriptase complex or other gRNA-interacting partners like the nucleocapsid protein (51, 52). The numerous structures found within the coding sequences of this region may also contribute to processes other than RNA synthesis, such as translational regulation (39, 53) and infectivity (19).

RNA folding is expected to be influenced by sequence context (54), and it is therefore notable that many structures predicted in downstream regions of the SARS-CoV-2 genome appear to depend on transcript positional context, as many of them occur at junctions between consecutive ORFs. Many potential structures will no longer form after leader-to-body fusion occurs at the junctional TRS sites (upon formation of an sgRNA), suggesting that certain structures may have roles only in the context of full-length genomic RNA and/or in longer sgRNAs that arise from upstream fusion events. We explored one example of such a structure (Fig. 7), which involves base pairings between a downstream segment of ORF8 and upstream segments of the N protein ORF, which are ablated upon formation of the N sgRNA. Genomic structures of this type may facilitate processes such as viral packaging (55) and may promote infectivity (56). On the other side of the spectrum, secondary structures that form exclusively in sgRNAs, such as the large motif predicted between the 5′ leader sequence and the N ORF (Fig. 7C), are expected to affect sgRNA properties like stability, abundance, and the recruitment of sgRNA-specific factors. Systematic structural comparisons among SARS-CoV-2 transcripts will certainly help to identify candidate genomic structures with potential roles in infectivity and to provide a framework for rationalizing the relative stabilities and functions of sgRNAs.

The vast genome of SARS-CoV-2 and its complex transcriptome present new challenges to RNA science, immunology, and medicine. However, the SARS-CoV-2 system and the intense attention it has attracted will also stimulate innovation, pushing researchers to develop new strategies for addressing the many challenges of studying and understanding exceptionally large RNA transcripts, particularly those in the life cycle of pathogens. We hope that the results and methods described in this work will provide a convenient roadmap to facilitate the design of new experiments for understanding the modular architecture of the SARS-CoV-2 genome and for unraveling the complex mechanisms of viral pathogenicity and host response. In addition, by focusing on the most structured regions of the genome and mapping their distribution, we seek to stimulate the search for promising new drug targets, thereby paving the way to novel therapeutic strategies against COVID-19 and other emerging RNA viruses.

## MATERIALS AND METHODS

### MFE Z-score analysis.

Folding minimum free-energy (MFE) Z-scores for SARS-CoV-2, HCV, West Nile virus (WNV), and human mRNAs (glyceraldehyde-3-phosphate dehydrogenase [GAPDH], beta-actin [ACTB], hypoxanthine phosphoribosyltransferase [HPRT], and α-tubulin) were calculated with the ScanFold program (57). ScanFold is a pipeline to scan and extract structural motifs from large RNA sequences (22), and it has recently been used to guide the design of small molecules targeting a SARS-CoV-2 frameshifting element (58). Part of the ScanFold suite, ScanFold-Scan uses ViennaRNA package 2.0 (59) to fold the target sequence in sliding windows and calculate the MFE secondary structure for each window. The Z-score for each window is computed by calculating by the difference between the native MFE and the average MFE of shuffled sequence controls and normalizing this difference by the standard deviation of the shuffled MFE distribution. ScanFold-Scan default parameters were used (120-nt window size, folding temperature of 37°C, mononucleotide shuffle procedure), with the exception of the number of randomizations (set to 100) and the sliding step size (set to 1 nt). Z-score frequency distributions for SARS-CoV-2, HCV, West Nile virus, and the composite of human mRNAs were calculated on GraphPad Prism.

### *In silico* RNA secondary structure modeling.

Secondary structure predictions for the full-length SARS-CoV-2 RNA genome, SARS-CoV-2 nucleocapsid (N) subgenomic RNA, and full-length HCV RNA genome were obtained using SuperFold with default settings as described by Smola et al. (29), which allow for reasonable computation times for long RNAs such as the viral genomes analyzed in this study. Since no experimental constraints were used in the modeling, the SHAPE contribution was canceled by setting both the SHAPE slope and intercept to 0 (–SHAPEslope 0; –SHAPEintercept 0). The default maximum base pairing distance (600 nt) was used in both partition function and windowed folding steps. Briefly, SuperFold calculates the base pairing partition function in 1,200-nt windows in steps of 100 nt along the RNA, while removing interactions occurring within the terminal 300 nt at the 5′ and 3′ ends of each window, yielding an effective window size of 600 nt (equivalent to the maximum base pairing distance) that is scanned across the full-length RNA; to compensate for the deweighting at the true 5′ and 3′ ends of the RNA, additional partition function calculations on those termini are performed. Base pairing probabilities are averaged across all windows in which a base pair is predicted to form. SuperFold then uses the Fold function from RNAstructure (60) to calculate the minimum free-energy secondary structure in 3,000-nt sliding windows every 300 nt along the RNA; highly probable base pairs from the partition function calculation (*P* > 99%) are used as hard constraints in this step. Finally, a consensus secondary structure is obtained by outputting base pairs consistently predicted during windowed folding, i.e., occurring in more than one-half of windows. The Shannon entropy for each nucleotide is computed with base pairing probabilities derived from the partition function calculation.

### BPC and BPC_rel_ calculations.

The base pair content (BPC) was calculated on Excel from the predicted secondary structure in sliding windows of 51 nt tiling the RNA in steps of 1 nt. For each nucleotide, we define BPC as the fraction of base-paired nucleotides within the window centered about that nucleotide. For the terminal 25 nucleotides at both ends of the RNA (window sizes < 51 nt), sliding windows were truncated accordingly. The BPC_rel_, i.e., the relative base pair content for a given nucleotide, was calculated with an in-house script by computing the percentile of the nucleotide’s absolute BPC among the global set of BPC values comprising the entire RNA. Briefly, 100,000 values were resampled with replacement (bootstrapping) from the set of BPC values. The bootstrapping is used as a convention to approximate the percentile of BPC values across a common denominator, regardless of the RNA tested. For each nucleotide, we then computed the fraction of bootstrapped values that lie below that nucleotide’s BPC score. By doing this, the median BPC value for a given RNA is “normalized” to 0.5, making for an intuitive measure of the relative significance of the magnitude of a given nucleotide’s BPC value. In case the median value itself is present multiple times in the BPC data set, the standardized median threshold shifts beyond 0.5, which can be corrected by resetting all median BPC values with a BPC_rel_ of 0.5. In this way, the rescaled BPC values (BPC_rel_) can be used for direct quantitative comparison of the structural content across any RNA.

### Identification of well-defined structures (high BPC/low Shannon).

BPC along with Shannon entropy values were used to identify nucleotides likely to form well-defined structures. In SHAPE experiments, this is accomplished by flagging those nucleotides with both low SHAPE reactivity and low Shannon entropy values, after smoothing both data sets in sliding windows tiling the RNA in steps of 1 nucleotide (29, 61). Nucleotides with SHAPE reactivities and Shannon entropy values below the global median of each respective distribution are considered highly structured and well defined. By analogy with the “low SHAPE/low Shannon” concept, here we define “high BPC/low Shannon” nucleotides as those likely engaged in well-defined structures. Shannon entropy values were first smoothed in 51-nt sliding windows (steps of 1 nt) to match the BPC calculation parameters. After computing BPC_rel_ values, nucleotides with BPC_rel_ greater than 0.5, i.e., above the global median of the distribution, and Shannon entropy values below the global median were flagged as high BPC/low Shannon. The fraction of well-defined structure in a given section of the RNA was then defined as the fraction of high-BPC/low-Shannon nucleotides in that section. For the comparison between *in silico* and experimental results for ORF1ab (Fig. 6), the high-BPC/low-Shannon distributions were computed from each individual structural model: the SHAPE-derived model reported by Huston et al. from infected cells (32) was used to generate the experimental distribution of well-defined structures across ORF1ab and compared against the *in silico* profile obtained in the present study for the same region (Fig. 4 and 6).

### Overlap between high-BPC/low-Shannon regions and regions with low average Z-scores.

It was important to assess the overlap between nucleotides in well-defined regions as defined by our approach (high BPC/low Shannon) and nucleotides with low average Z-scores (Z_avg_) from the ScanFold-Fold analysis of SARS-CoV-2 reported by Andrews et al. (13). To this end, Z_avg_ scores for SARS-CoV-2 were downloaded from the Moss lab RNAStructuromeDB (https://structurome.bb.iastate.edu/). In order to match the criteria we used to flag well-defined structures (high BPC/low Shannon, both relative to the global median), low Z_avg_ values are defined here as those occurring below the distribution median of Z_avg_ values, i.e., regions with folding stability above the average. The statistical significance of the overlap between both methods was evaluated on MATLAB by running simulations of randomly distributed elements in both groups. The *P* value was estimated by computing the number of times an overlap equal or greater than the observed value was obtained and then dividing it by the number of simulations.

### Nucleocapsid sgRNA SHAPE-MaP probing and structure modeling.

SARS-CoV-2 infection in Vero cells, NAI SHAPE probing, and SHAPE-MaP library preparation were performed exactly as described by Huston et al. (32). The following strategy was used to specifically target the nucleocapsid sgRNA: reverse transcription (RT) was performed with MarathonRT (62) using a primer targeting the 3′ end of the SARS-CoV-2 3′ UTR (5′-TTTTTTTTTGTCATTCTCC-3′); cDNA was then amplified with a forward primer targeting the 5′ end of SARS-CoV-2 5′-leader sequence (5′-ATTAAAGGTTTATACCTTCCCAG-3′) and a reverse primer targeting the 3′ end of the SARS-CoV-2 3′ UTR (5′-TTTTTTGTCATTCTCCTAAGAAG-3′). In conjunction with a short PCR extension time (2 min), this RT-PCR design allows for selective amplification of the N sgRNA sequence and cannot efficiently amplify intervening regions in the gRNA or other sgRNAs. To verify selective amplification of the N sgRNA, the correct amplicon size (1,686 bp) was confirmed by gel electrophoresis. Libraries were sequenced on the Illumina NextSeq 500/550 platform using a 2 × 75-bp paired-end sequencing. Sequencing data were analyzed using the ShapeMapper 2 analysis pipeline (63), aligning reads to the N sgRNA sequence. To ensure that sequencing reads corresponded to the N sgRNA, alignments generated with the ShapeMapper 2 were visualized on IGV (v2.8.2) to confirm the presence of high-quality chimeric reads that span the TRS junction site between the leader sequence and the nucleocapsid ORF. SuperFold (29) was then used to model the secondary structure of the N sgRNA using in-cell SHAPE reactivities and the same modeling parameters as described by Huston et al. (32). The experimental secondary structure model for the SARS-CoV-2 genomic RNA previously reported (32) was used for comparison of the N ORF RNA folding between genomic and subgenomic contexts.

### Reference sequences.

The SARS-CoV-2 reference genome from Wu et al. (1) was used for all analyses, along with the protein annotations deposited in NCBI (GenBank accession numbers MN908947.3 and NC_045512.2). Human beta-actin mRNA (NM_001101.5), human GAPDH mRNA (NM_002046.7), human HPRT mRNA (NM_000194.3), human α-tubulin 1 mRNA (NM_006009.4), West Nile virus genome (NY99 sequence, based on DQ211652.1 reference) and HCV genome (JC1 sequence, based on JF343782.1 reference) sequences were used for Z-score analysis.

### Data availability.

Data sheets, sequence files, and the script used to calculate relative BPC values are available at the GitHub repository: https://github.com/pylelab/SARS-CoV-2_global_local_structure.
